# Parameter Optimization of Model Predictive Direct Motion Control for Distributed Drive Electric Vehicles Considering Efficiency and the Driving Feeling

**DOI:** 10.3390/s23146324

**Published:** 2023-07-12

**Authors:** Lixiao Gao, Feng Chai

**Affiliations:** School of Electrical Engineering and Automation, Harbin Institute of Technology, Harbin 150001, China; chaifeng@163.com

**Keywords:** parameter optimization, distributed drive electric vehicles, PMSM, overall efficiency, driving feeling, LSTM neural network

## Abstract

This paper presents a novel motion control strategy based on model predictive control (MPC) for distributed drive electric vehicles (DDEVs), aiming to simultaneously control the longitudinal and lateral motion while considering efficiency and the driving feeling. Initially, we analyze the vehicle’s dynamic model, considering the vehicle body and in-wheel motors, to establish the foundation for model predictive control. Subsequently, we propose a model predictive direct motion control (MPDMC) approach that utilizes a single CPU to directly follow the driver’s commands by generating voltage references with a minimum cost function. The cost function of MPDMC is constructed, incorporating factors such as the longitudinal velocity, yaw rate, lateral displacement, and efficiency. We extensively analyze the weighting parameters of the cost function and introduce an optimization algorithm based on particle swarm optimization (PSO). This algorithm takes into account the aforementioned factors as well as the driving feeling, which is evaluated using a trained long short-term memory (LSTM) neural network. The LSTM network labels the response under different weighting parameters in various working conditions, i.e., “Nor”, “Eco”, and “Spt”. Finally, we evaluate the performance of the optimized MPDMC through simulations conducted using MATLAB and CarSim software. Four typical scenarios are considered, and the results demonstrate that the optimized MPDMC outperforms the baseline methods, achieving the best performance.

## 1. Introduction

Vehicle motion control is primarily dependent on the manipulation of steering angles and torque applied to the wheels. These parameters are determined by sophisticated path planning systems [1,2]. To ensure both longitudinal movement control and stability during lateral movement, wheel torques must be carefully regulated [3]. Among various vehicle configurations, distributed drive electric vehicles (DDEVs) have attracted significant attention due to their unique ability to independently control the torques of all four wheels. This feature provides substantial advantages in terms of longitudinal, lateral, and yaw motion control [4,5].

The existing motion control systems for DDEVs typically consist of a vehicle handling controller and four motor controllers [6,7]. The vehicle handling controller’s main objective is to accurately track movement references in different directions for DDEVs. However, it faces several challenges that need to be addressed. These challenges include the nonlinearity of the vehicle mathematical model, constraints imposed by the system, and the over-actuation problem [8,9].

The longitudinal motion control of DDEVs is commonly achieved by manipulating the acceleration and brake pedals. Recent research has focused on integrating real-time road conditions and vehicle dynamics to make comprehensive judgments about the optimal longitudinal speed. This approach aims to enhance both passenger comfort and fuel economy [10,11]. Notably, a literature study [12] proposed a vehicle speed control strategy based on road gradient information with the objective of reducing energy consumption. This strategy effectively reduces energy usage while improving computational efficiency.

Furthermore, various control strategies have been developed to improve the stability and handling of DDEVs. For instance, Ref. [13] addressed the steering stability control of four in-wheel motor drive electric vehicles on a road with varying adhesion coefficients. Another study [14] designed a sliding mode direct yaw moment control system specifically for in-wheel electric vehicles. Moreover, Ref. [15] proposed a post-impact stability control method for four hub motor independent-drive electric vehicles.

Model predictive control (MPC) has been extensively applied in the control of DDEVs. Researchers have developed a real-time nonlinear model predictive controller for optimizing the yaw motion of distributed drive electric vehicles [16]. Another study proposed a path tracking and direct yaw moment coordinated control system based on robust MPC for autonomous independent-drive vehicles [17]. Additionally, a nonlinear model predictive lateral stability control system for the active chassis of intelligent vehicles was designed in [18].

Several studies have also investigated the integration of longitudinal, lateral, and vertical vehicle stability control for DDEVs. For example, Ref. [19] proposed a real-time NMPC strategy that combines torque vectoring with rear wheel steering to improve electric vehicle stability. In another study, an analysis of integrated longitudinal and lateral vehicle stability control was performed under extreme conditions with safety dynamic requirements [20]. Additionally, a handling and stability integrated control system based on risk assessments and predictions was developed in [21]. Moreover, Ref. [22] proposed a multi-level coordinated yaw stability control strategy based on sliding mode predictive control for DDEVs under extreme conditions. Lastly, Ref. [23] presented a cooperative strategy for trajectory tracking and stability control.

However, none of the aforementioned literature is based on the MPDMC framework. An additional controller is required to interpret driver control commands, convert throttle signals into torque commands for different hub motors, and then achieve torque tracking through dedicated motor controllers. In this framework, the two-level controllers directly exchange limited information through specific communication methods. Moreover, communication between the two levels of controllers introduces certain delays and some computations between the two levels of controllers are redundant, which adversely affects the system’s fast response. Furthermore, such a structure requires two separate controllers and related peripheral hardware, increasing the cost of the vehicle.

Taking into account the significant improvements in microprocessor computing speeds, this paper proposes a novel approach to MPDMC, utilizing a single controller to achieve motion control in all three directions of the DDEV. This strategy uses the longitudinal velocity, sideslip angle, and yaw rate of the vehicle as inputs, and combinations of voltage vectors for the in-wheel motor drive systems are produced as outputs. By employing MPDMC, the DDEV motion is directly controlled.

In this study, a unified mathematical model of the vehicle and motors is established. Due to the distinct time constants of the vehicle system and the motor system, the coupling relationship between the two systems during the prediction process is carefully analyzed, along with a detailed examination of the simplified method.

Furthermore, a cost function is designed, which incorporates tracking the expected value and satisfying constraint conditions. The longitudinal velocity, yaw rate, lateral displacement, and efficiency are introduced into the cost function with weighting parameters. The candidate voltage vector combination that minimizes the cost function is determined as the final action to be implemented on the motor inverters.

To address the issue of a large number of candidate voltage vector combinations for the four motors, as well as the repetition of voltage vector combinations resulting from the over-actuation problem, the concept of deadbeat control is introduced. A torque distribution method considering the vertical loads of each wheel is employed to simplify the candidate voltage vector combinations. By adopting this MPDMC approach, the proposed control system achieves efficient and precise control over the DDEVs motion, while also considering the driving experience. The integration of control tasks into a single controller not only eliminates the need for additional controllers and peripheral hardware, reducing the vehicle’s cost, but also improves the system’s responsiveness and computational efficiency.

The weight coefficients associated with different terms in the cost function have a significant impact on the final control results. However, due to the complexity of the controlled system, selecting appropriate weight coefficients is a challenging problem. To address this issue, the particle swarm optimization (PSO) algorithm is introduced in this work to optimize the weight coefficients.

Similarly, the optimization process requires the introduction of a new cost function to evaluate the effectiveness of different weight coefficients. In addition to the conventional control terms mentioned earlier, this paper introduces a fuzzy evaluation term called “driving feeling”. This term is evaluated using a long short-term memory (LSTM) neural network. The vehicle response curves under different driving styles are fed into the LSTM network for training.

During the PSO optimization process, the response curves of different weight coefficients are classified by the trained LSTM network to account for various driving experiences. By incorporating the optimization of weight coefficients using the PSO algorithm and considering the fuzzy evaluation of driving feeling through the trained LSTM network, the proposed approach aims to enhance both the efficiency and driving experience of the DDEVs motion control. The optimized weight coefficients contribute to achieving desired control performance, while the consideration of driving feeling adds a human-centric aspect to the control system. This integration of optimization and subjective evaluation provides a more comprehensive and tailored control strategy for the DDEV, further improving its overall performance.

In this paper, we offer a simplified control structure by replacing multiple controllers with a single controller, enhancing the system handling quality. The primary objective of the MPDMC approach is to simultaneously control the longitudinal and lateral motion of the vehicle while considering efficiency and the driving feeling. The main contributions of this article can be summarized as follows:

- **Introduction of a simplified control structure:** The MPDMC strategy replaces the traditional motion control system, which typically requires one vehicle handling controller and four motor controllers, with a streamlined control structure that integrates the control of three motion directions into a single controller. This simplification improves the handling quality of the system and reduces the system complexity.
- **Optimization considering efficiency and driving feeling:** The paper presents a comprehensive cost function that balances control accuracy and efficiency. This cost function takes into account various control objectives and constraint conditions. Additionally, the integration of a subjective driving feeling evaluation, achieved through a trained LSTM neural network, allows for a more balanced and tailored control strategy. The optimization process, utilizing the particle swarm optimization (PSO) algorithm, determines the weight parameters associated with different terms in the cost function, enhancing the overall control performance.

This article is organized as follows. Section 2 presents a unified mathematical model of DDEV. Then, the proposed MPDMC strategy is detailed in Section 3. Furthermore, Section 4 details the construction of the cost function of MPDMC. Section 5 focuses on the cost functions considering driving feeling using an LSTM neural network. Section 6 introduces the PSO optimization of MPDMC considering the driving feeling through LSTM. Finally, the proposed algorithm is evaluated in Section 7 and Section 8 concludes this paper.

## 2. Model of DDEV

### 2.1. Dynamics Model

**Figure 1:**
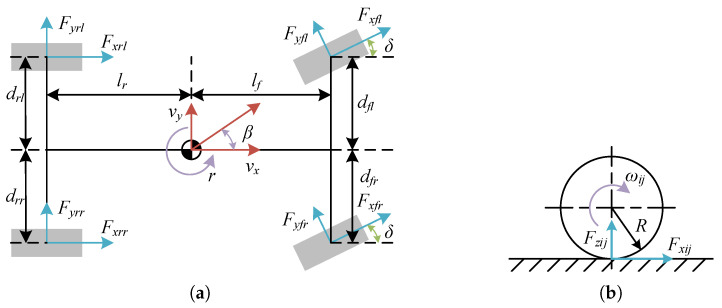
Dynamics model. (**a**) Vehicle dynamics model. (**b**) Wheel dynamics model.

The dynamics model is shown in Figure 1 and encompasses three fundamental components: longitudinal movement, lateral movement, and yaw movement. Each of these components contributes to the overall motion of the vehicle body on the plane. The dynamics equations governing these movements are outlined below:

Longitudinal Movement:

(1) $$\begin{array}{cc} m\frac{dv_{x}}{dt}= & mv_{y}r+\cos\delta \left(F_{xfl}+F_{xfr}\right)\\ \; & -\sin\delta \left(F_{yfl}+F_{yfr}\right)+F_{xrl}+F_{xrr} \end{array}$$

Lateral movement:

(2) $$\begin{array}{cc} m\frac{dv_{y}}{dt}= & -mv_{x}r+\sin\delta \left(F_{xfl}+F_{xfr}\right)\\ \; & +\cos\delta \left(F_{yfl}+F_{yfr}\right)+F_{yrl}+F_{yrr} \end{array}$$

Yaw movement:

(3) $$\begin{array}{cc} J_{z}\frac{dr}{dt}= & \left(-d_{fl}\cos\delta +l_{f}\sin\delta \right)F_{xfl}\\ \; & +\left(d_{fr}\cos\delta +l_{f}\sin\delta \right)F_{xfr}\\ \; & -d_{rl}F_{xrlr}+d_{rr}F_{rr}+l_{f}\cos\delta \left(F_{yfl}+F_{yfr}\right)\\ \; & +d_{fl}\sin\delta F_{yfl}-d_{fr}\sin\delta F_{yfr}-l_{r}\left(F_{yrl}+F_{yrr}\right) \end{array}$$

where $v_{x}$, $v_{y}$, and *r* are the longitudinal speed, lateral speed, and yaw rate of DDEV; $F_{xfl},F_{xfr},F_{xrl}$, and $F_{xrr}$ are the longitudinal forces experienced by the front left wheel, front right wheel, rear left wheelm and rear right wheel, respectively; $F_{yfl},F_{yfr},F_{yrl}$, and $F_{yrr}$ are the lateral tire forces of the four wheels; δ is the front steering angle; *m* is the vehicle mass; $l_{f}$ and $l_{r}$ are the distances from the center of gravity to the front and rear axles; and $J_{z}$ is the yaw moment of inertia of the vehicle. To simplify the model, we omit the consideration of forces resisting motion, such as aerodynamic forces or rolling friction, converting such forces to the traction torque of the four wheels. This simplification is made to streamline the motion model and focus on the core aspects of the proposed MPDMC strategy. If a more comprehensive representation of the vehicle dynamics is desired, it is indeed possible to introduce these forces into the motion model. Incorporating these factors would make the model predictive control more accurate, albeit at the cost of an increased complexity.

### 2.2. In-Wheel Motor Model

The dynamic model of the in-wheel motor is as follows:

(4) $$J_{w}\frac{\omega_{ij}}{dt}=T_{eij}-RF_{xij}$$

where i=f,r is the front and rear wheel, j=l,r is the left and right wheel, $T_{eij}$ is the electric torque generated by the four in-wheel motors, $J_{w}$ is the inertia of the wheel, *R* is the radius of the wheel, and $\omega_{ij}$ is the wheel speed.

This study focuses on utilizing permanent magnet synchronous motors (PMSMs) as the in-wheel motors due to their numerous advantages. PMSMs offer a high power density and efficiency, a precise torque control, a wide speed range and reliability, and compatibility with advanced control techniques. These qualities make PMSMs an ideal choice for exploring and evaluating control strategies in electric motor systems. The voltage and torque model at the d-q axis is as follows:

(5) $$\left\{\begin{array}{c} \;\frac{di_{dij}}{dt}=-\frac{R_{s}}{Ld}i_{dij}+\omega_{eij}i_{qij}+\frac{u_{dij}}{Ld}\\ \frac{di_{qij}}{dt}=-\frac{R_{s}}{Ld}i_{qij}-\omega_{eij}i_{dij}+\frac{u_{qij}}{Lq}-\frac{\psi_{f}}{Lq}\omega_{eij} \end{array}\right.$$

(6) $$T_{eij}=3/2p\psi_{f}i_{qij}$$

where $u_{dij}$, $i_{dij}$ and $u_{qij}$, $i_{qij}$ are the d- and q-axis stator voltages and currents of ij-th motor, respectively; *p* is the number of poles; $R_{s}$, $L_{d}$, and $L_{q}$ are the stator resistance and the dq axis inductance; and $\psi_{f}$ is the magnetic flux linkage.

The relationship between $T_{eij}$ and $F_{xij}$ can be approximated as:

(7) $$T_{eij}=F_{xij}R$$

### 2.3. Unified Model

Combining the mathematical models of the vehicle and motors, one can obtain the unified mathematical model:

(8) $$\left\{\begin{array}{c} \dot{x}=f\left(x,u\right)\\ y=h(x) \end{array}\right.$$

where $x={\left[x_{1}|x_{2}\right]}^{T}={\left[v_{x}\;v_{y}\;r|\;i_{\mathrm{dij}}\;i_{\mathrm{qij}}\right]}^{T}$ is a 11 × 1 state variable matrix; $u={\left[u_{\mathrm{dij}}|u_{\mathrm{qij}}\right]}^{T}$ is a 8 × 1 control variable matrix; and $y={\left[y_{1}|y_{2}\right]}^{T}={\left[v_{x}\;v_{y}\;r|\;i_{\mathrm{dij}}\right]}^{T}$ is a 7 × 1 output variable matrix.

## 3. Proposed MPDMC Strategy

**Figure 2:**
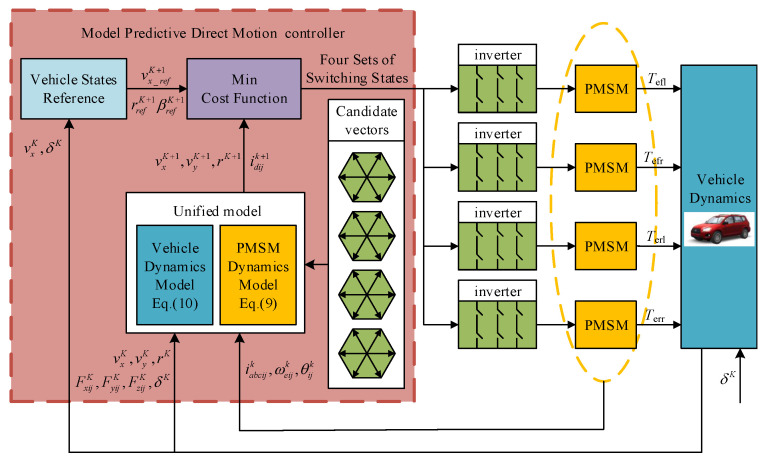
The proposed MPDMC strategy.

A novel strategy called MPDMC is introduced in this paper, which has the capability to predict future vehicle body states within a limited time frame and generate the optimal voltage vector combination for in-wheel motor drive systems. Figure 2 illustrates the structure of the MPDMC strategy.

### 3.1. Reference Inputs

The reference inputs of the system are driving commands from the driver, including the longitudinal speed $v_{xref}$ and the steering angle σ. The proposed motion control strategy, MPDMC, aims to control the longitudinal and lateral motion of the vehicle simultaneously. To clarify, when a driver wants to steer the vehicle, they turn the steering wheel, which results in a corresponding steering angle σ. There are two aspects involved in the steering process. Firstly, the mechanical structure of the vehicle associates each steering angle with a corresponding steering angle for the front wheels. This mechanical relationship ensures the vehicle steers in response to the driver’s input. Secondly, the steering angle σ is fed into the MPDMC controller. Through a torque distribution algorithm, the MPDMC controller calculates the desired reference torques for each of the four wheels. These reference torques are then used to control the four-wheel torques by adjusting the applied voltages using the MPDMC strategy. This control process ensures that the vehicle turns with a low slip rate and tire slip rate, optimizing the steering performance.

### 3.2. Discretization of the Mathematical Model

To implement the MPDMC system on a microprocessor, it is necessary to discretize the unified continuous mathematical model of the vehicles and motors. The vehicle discretization sampling time, denoted as $T_{sv}$, should be chosen as an integer multiple of the motor discretization sampling time $T_{sm}$, such that $T_{sv}=nT_{sm}$ (where *n* is an integer). The motor voltage equation is discretized using the forward Euler method:

(9) $$\left\{\begin{array}{cc} \;i_{dij}^{k+1}= & \left(1-\frac{R_{s}T_{sm}}{L_{d}}\right)i_{dij}^{k}+T_{sm}i_{qij}^{k}\omega_{eij}^{k}+\frac{R_{s}T_{sm}}{L_{d}}u_{dij}^{k}\\ \;i_{qij}^{k+1}= & \left(1-\frac{R_{s}T_{sm}}{L_{q}}\right)i_{qij}^{k}-T_{sm}i_{dij}^{k}\omega_{eij}^{k}\\ \; & -\frac{\psi_{f}T_{sm}}{L_{q}}\omega_{eij}^{k}+\frac{T_{sm}}{L_{q}}u_{dij}^{k} \end{array}\right.$$

where the superscript *k* represents the *k*th instant in the motor time domain.

(10) $$\left\{\begin{array}{c} \;m\frac{\Delta v_{x}\left(K\right)}{T_{sv}}=mv_{y}\left(K\right)r\left(K\right)+\cos\delta \left(F_{xfl}\left(K\right)+F_{xfr}\left(K\right)\right)\\ \;\;-\sin\delta \left(F_{yfl}\left(K\right)+F_{yfr}\left(K\right)\right)+F_{xrl}\left(K\right)+F_{xrr}\left(K\right)\\ \;m\frac{\Delta v_{y}\left(K\right)}{T_{sv}}=-mv_{x}\left(K\right)r\left(K\right)+\sin\delta \left(F_{xfl}\left(K\right)+F_{xfr}\left(K\right)\right)\\ \;\;+\cos\delta \left(F_{yfl}\left(K\right)+F_{yfr}\left(K\right)\right)+F_{yrl}\left(K\right)+F_{yrr}\left(K\right)\\ \;J_{z}\frac{\Delta r(K)}{T_{sv}}=\left(-d_{fl}\cos\delta +l_{f}\sin\delta \right)F_{xfl}\left(K\right)+F_{xfr}\left(K\right)\\ \;\;+\left(d_{fr}\cos\delta +l_{f}\sin\delta \right)-d_{rl}F_{xrlr}\left(K\right)+d_{rr}F_{rr}\left(K\right)\\ \;\;+l_{f}\cos\delta \left(F_{yfl}+F_{yfr}\right)-l_{r}\left(F_{yrl}\left(K\right)+F_{yrr}\left(K\right)\right)\\ \;+d_{fl}\sin\delta F_{yfl}\left(K\right)-d_{fr}\sin\delta F_{yfr}\left(K\right) \end{array}\right.$$

where the superscript *K* represents the *K*th instant in the motor time domain, $\Delta v_{x}\left(K\right)=v_{x}\left(K+1\right)-v_{x}\left(K\right)$, $\Delta v_{y}\left(K\right)=v_{y}\left(K+1\right)-v_{y}\left(K\right)$, and Δr(K)=r(K+1)−r(K).

**Figure 3:**
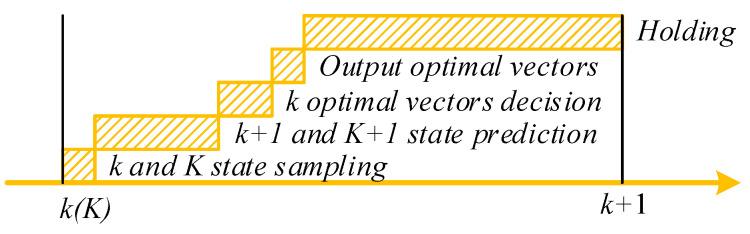
Prediction process at the kth instant.

The specific timing diagram of MPDMC is shown in Figure 3. Taking the moment *t* in the motor time domain as an example, firstly, the dq-axis current and electrical angular velocity of the motor at time *k*, as well as the longitudinal velocity, lateral velocity, and yaw rate of the vehicle at time *K*, are collected. Then, based on the candidate voltage vector combinations, the dq-axis current of the four wheel hub motors at time *k* + 1, as well as the longitudinal velocity, lateral velocity, and yaw rate at time *K* + 1, are predicted. Subsequently, using these predicted values and their corresponding target values, an evaluation function is designed to select the voltage vector combination that minimizes the evaluation function value, which is then output to the inverter. The following sections will provide detailed explanations of the evaluation function design and the selection of candidate voltage vector combinations.

### 3.3. Inverter Voltage Vectors

For a three-phase voltage source inverter of the ijth wheel, the output voltages of three motor phases, referred to as their star point, can be expressed as:

(11) $$U_{abc}=\left[\begin{array}{c} U_{a}\;U_{b}\;U_{c} \end{array}\right]=\frac{U_{dc}}{3}\left[\begin{array}{ccc} 2 & -1 & -1\\ -1 & 2 & -1\\ -1 & -1 & 2 \end{array}\right]\left[\begin{array}{c} S_{a}\;S_{b}\;S_{c} \end{array}\right]$$

where $U_{abc}={\left[U_{a}\;U_{b}\;U_{c}\right]}^{T}$ represents the phase voltages at the terminals and ${\left[S_{a}\;S_{b}\;S_{c}\right]}^{T}$ represents the states of the three-phase inverter switches. There are a total of $2^{3}$ combinations: ${\left[0\;0\;0\right]}^{T}$, ${\left[0\;0\;1\right]}^{T}$, ${\left[0\;1\;0\right]}^{T}$, ${\left[0\;1\;1\right]}^{T}$, ${\left[1\;0\;0\right]}^{T}$, ${\left[1\;0\;1\right]}^{T}$, ${\left[1\;1\;0\right]}^{T}$, ${\left[1\;1\;1\right]}^{T}$. $U_{dc}$ represents the bus voltage.

## 4. Cost Functions of MPDMC

To achieve the accurate and stable operation of DDEV systems, the control objectives of the vehicle can be divided into three main parts. Firstly, the longitudinal velocity is chosen as the control objective to ensure the vehicle’s longitudinal motion. Secondly, to stabilize the vehicle’s control, the yaw rate and the lateral deviation angle of the center of mass are typically used as control objectives. Based on the two-degree-of-freedom vehicle model, the desired values for the yaw rate and the lateral deviation angle of the center of mass can be obtained. Thirdly, in order to increase the electric vehicle’s driving range, we have included a loss term in the cost function to evaluate the power losses at the next time step for different voltage vector combinations.

### 4.1. Longitudinal Velocity Control Objective

In order to accurately control the velocity of a vehicle, the cost function of the longitudinal velocity is defined as:

(12) $$C_{v}=\left|\right|v_{xref}^{K+1}-v_{x}^{K+1}{\left|\right|}^{2}$$

The longitudinal velocity of a vehicle is controlled through the output torque of the four in-wheel motors, which is generated under different voltage vectors.

### 4.2. Stability of the Vehicle

To achieve stable operation of DDEV systems, the given values for the yaw rate and the lateral displacement of the center of mass can be obtained from the two-degree-of-freedom vehicle model. The values of $r_{ref0}$ and $\beta_{ref0}$ for DDEV are determined by calculating the steady state of a two DOF linear bicycle model, which focuses solely on tire lateral forces and utilizes a linear model [8,14,24]:

(13) $$r_{ref0}^{K+1}=\frac{v_{x}^{K}}{\left(l_{f}+l_{r}\right)\left(1+r{\left(v_{x}^{K}\right)}^{2}\right)}\delta^{K}$$

(14) $$\beta_{ref0}^{K+1}=\frac{l_{r}-\frac{l_{f}m{\left(v_{x}^{K}\right)}^{2}}{2c_{f}\left(l_{f}+l_{r}\right)}}{\left(l_{f}+l_{r}\right)\left(1+\beta_{1}{\left(v_{x}^{K}\right)}^{2}\right)}\delta^{K}$$

where $\beta_{1}=m\left(l_{r}c_{r}-l_{f}c_{f}\right)/\left(2c_{f}c_{r}{\left(l_{f}+l_{r}\right)}^{2}\right)$ and $c_{f}$ and $c_{r}$ represent the lateral stiffness of the front and rear tires, respectively. Taking into account the conditions of the tires and road surface, the constraint conditions for the yaw rate and lateral displacement of the center of mass are as follows:

(15) $$r_{ref}^{K+1}=\left\{\begin{array}{cc} r_{ref0}^{K+1} & |r_{ref0}^{K+1}|\leq r_{max}\\ r_{max}sign\left(r_{ref0}^{K+1}\right) & |r_{ref0}^{K+1}|>r_{max} \end{array}\right.$$

(16) $$\beta_{ref}^{K+1}=\left\{\begin{array}{cc} \beta_{ref0}^{K+1} & |\beta_{ref0}^{K+1}|\leq \beta_{max}\\ \beta_{max}sign\left(\beta_{ref0}^{K+1}\right) & |\beta_{ref0}^{K+1}|>\beta_{max} \end{array}\right.$$

where $r_{max}=0.85\;\mu g/v_{x}^{K}$, $\beta_{max}=arttan\left(0.02\;\mu g\right)$ is the limit of the yaw rate and lateral displacement, μ is the road adhesion coefficient, and *g* is gravitational acceleration.

In this way, the cost function of the stability of a vehicle consists of the yaw rate control error and the lateral deviation angle:

(17) $$C_{r}=\left\{\begin{array}{cc} \left|\right|r_{ref}^{K+1}-r^{K+1}{\left|\right|}^{2} & |r^{K+1}|\leq r_{max}\\ inf & |r^{K+1}|>r_{max} \end{array}\right.$$

(18) $$C_{\beta }=\left\{\begin{array}{cc} \left|\right|\beta_{ref}^{K+1}-\beta^{K+1}{\left|\right|}^{2} & |\beta^{K+1}|\leq \beta_{max}\\ inf & |\beta^{K+1}|>\beta_{max} \end{array}\right.$$

The condition in the second line is used to prevent the variables $r^{K+1}$ and $\beta^{K+1}$ from exceeding the specified limits ($r_{max}$ and $\beta_{max}$).

### 4.3. Efficiency of DDEVs

In this paper, we focus on the power loss of the inverter and the motor, discarding other power losses such as those of the battery and transmission system. It is worth noting that we do not propose power loss optimal motor control methods. There is a number of previous works providing extensive algorithms to minimize the power loss of motor control systems. We introduce such information to enhance the MPDMC performance.

#### 4.3.1. Power Loss of the Inverter

Various methods are described in the scientific literature for evaluating power electronic converter losses [25,26] and establishing an appropriate energy model. The simplest approach is to treat the power converter as an equivalent resistive load, where the internal power losses are proportional to the square of the current flowing through it. As most power converters adopt a three-phase inverter topology, the expression for the power loss can be formulated as $P_{LossInv}=3R_{inv}I^{2}$, where $R_{inv}$ represents the equivalent resistance of the inverter and $I=\sqrt{i_{d}^{2}+i_{q}^{2}}$ denotes the instantaneous current of the inverter output phase (equivalent to the RMS current of the input electromagnetic phase).

#### 4.3.2. Power Loss of the PMSM

**Figure 4:**
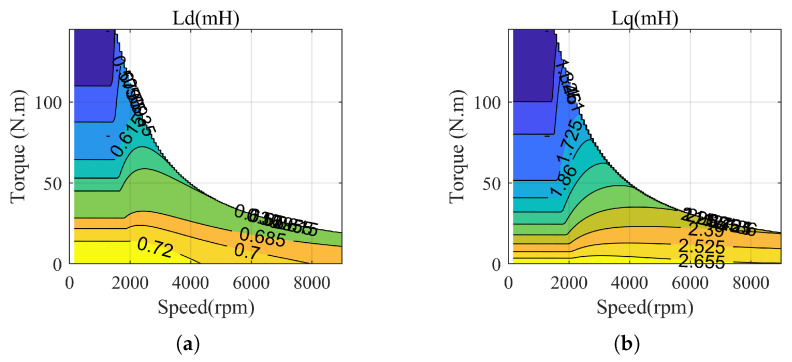
Dynamic parameters map. (**a**) D-axis inductance. (**b**) Q-axis inductance.

The loss analysis of the PMSM is a challenging problem. Simply put, motor losses are mainly composed of copper losses and iron losses, which have been extensively discussed in numerous papers [27,28,29]. However, the actual motor losses are influenced by more complex factors. On the one hand, the parameters of the motor change dynamically during operation with variations in current and temperature. The specific static parameters of the motor used in this paper are shown in Table 1. During dynamic operation, the dq-axis inductance undergoes certain changes, as illustrated in Figure 4. On the other hand, the harmonics, friction loss in the bearings, and winding loss effects are all aspects that affect the power loss [30].

#### 4.3.3. Maximum Efficiency Operating Map

**Figure 5:**
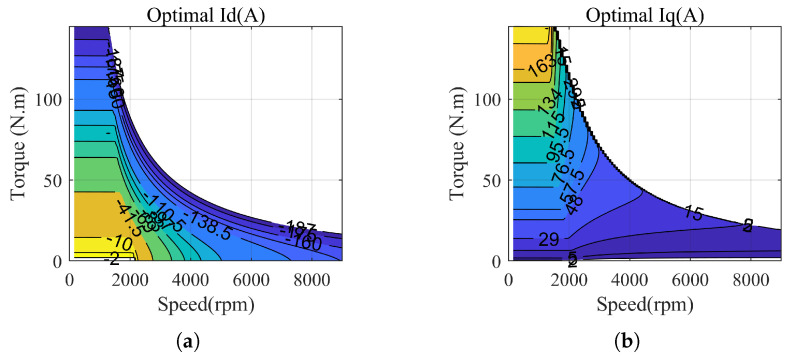
Optimal operating dq-axis current with maximum efficiency. (**a**) Optimal Id. (**b**) Optimal Iq.

**Figure 6:**
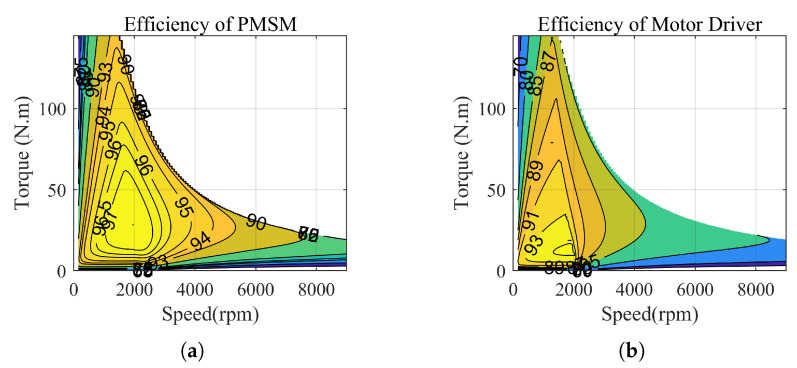
Efficiency map. (**a**) Efficiency of PMSM. (**b**) Efficiency of motor driver.

Due to the aforementioned reasons and limitations in terms of space, the efficiency operating chart of the motor is directly presented in this paper. Under specific operating conditions, the optimal given values of dq-axis currents are shown in Figure 5. When the speed exceeds the rated speed, motor weakening techniques are typically employed. However, the specific optimization of PMSM operation is beyond the scope of this paper. In Figure 5, we consider the weakening and power loss to obtain the optimal Id. Due to space limitations, we are unable to provide a detailed analysis in this regard. The efficiency chart of the motor and the efficiency chart of the motor with an inverter are depicted in Figure 6. Under different operating conditions, the given values of q-axis currents are calculated by the model, then the optimal d-axis currents $i_{dopt}$ are obtained directly through consulting the table. The corresponding cost function for power losses is as follows:

(19) $$C_{l}=\left\{\begin{array}{cc} \left|\right|i_{dopt}^{K+1}-i_{d}^{K+1}{\left|\right|}^{2} & sqrt\left({\left(i_{d}^{K+1}\right)}^{2}+{\left(i_{q}^{K+1}\right)}^{2}\right)\leq I_{max}\\ inf & sqrt\left({\left(i_{d}^{K+1}\right)}^{2}+{\left(i_{q}^{K+1}\right)}^{2}\right)>I_{max} \end{array}\right.$$

The condition in the second line is used to prevent the current from exceeding the maximum current $I_{max}$.

#### 4.3.4. Entire Cost Function of MPDMC

**Figure 7:**
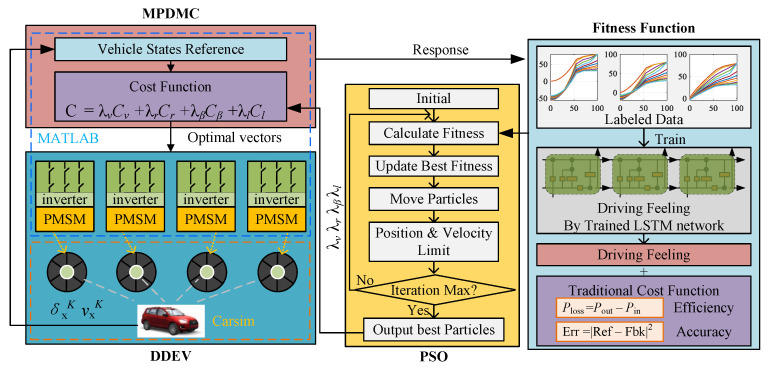
The optimization process based on PSO.

Based on the above factors, the entire cost function of MPDMC is constructed as their sum:

(20) $$C=\lambda_{v}C_{v}+\lambda_{r}C_{r}+\lambda_{\beta }C_{\beta }+\lambda_{l}C_{l}$$

where $\lambda_{v}$, $\lambda_{r}$, $\lambda_{\beta }$, and $\lambda_{l}$ represent the weighting coefficients for longitudinal velocity, steering angle, yaw angle, and losses, respectively. These coefficients directly influence the selection of the optimal voltage vector in MPDMC. A higher value of the corresponding weighting coefficient increases the impact of the respective term during model prediction. However, overweighting coefficients can lead to an insufficient influence from other terms. Therefore, it is necessary to balance these coefficients in order to achieve the best overall control (Figure 7).

## 5. Optimization of Cost Functions Considering Driving Feeling

In this section, we will introduce the evaluation of driving feeling using an LSTM neural network.

### 5.1. Typical Labeled Data

**Figure 8:**
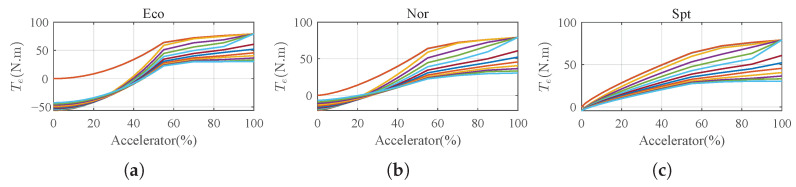
Typical required torque vs. acceleration with driving feeling labeled as “Eco”, “Nor”, and “Spt”. (**a**) Labeled ’Eco’. (**b**) Labeled ’Nor’. (**c**) Labeled ’Spt’.

The driving feeling of the driving environment is a critical aspect of autonomous vehicles, as it involves understanding and interpreting the surrounding objects, road conditions, and potential hazards. Traditional approaches to evaluating the driving feeling often rely on rule-based systems or handcrafted features, which may have limitations in capturing complex and dynamic driving scenarios. In this paper, we focus on the driving feeling associated with the acceleration of longitudinal velocity. The requirement for electric torque for each in-wheel motor at different initial speeds is shown in Figure 8. To generate the curves, an experimental setup was designed to collect data on various driving scenarios and conditions. After collecting the data, a group of consumers participated in labeling the driving feelings based on their subjective perceptions and experiences. They evaluated the driving characteristics and provided feedback on the corresponding torque references for each driving feeling. In each figure, there are 13 curves for the initial speeds of 0, 10, 20, …, 120 km/h marked in different colors. There are three typical driving modes that can be referred to as “ecology conservation optimization”, “normal”, and “sport”, commonly abbreviated as “Eco”, “Nor”, and “Spt”, respectively. These driving modes represent different preferences in terms of vehicle performance and efficiency. The “Eco” mode focuses on maximizing fuel efficiency and minimizing the environmental impact. The “Nor” mode represents a balanced driving style that is suitable for everyday driving conditions. The “Spt” mode emphasizes a more dynamic and sporty driving experience, with increased responsiveness and performance. These driving modes allow drivers to select a preferred driving style based on their individual preferences and driving conditions.

The purpose of training a neural network in this study is for it to serve as a fuzzy cost function to evaluate the performance of the proposed motion control strategy under different weighting parameters. This allows the network to classify and evaluate the driving performance based on the given input parameters. By incorporating the trained neural network as a fuzzy cost function, the proposed motion control strategy can adapt its control decisions based on the desired driving feeling. The weighting parameters in the cost function can be adjusted to prioritize certain driving modes or optimize specific performance criteria.

### 5.2. LSTM Training

**Figure 9:**
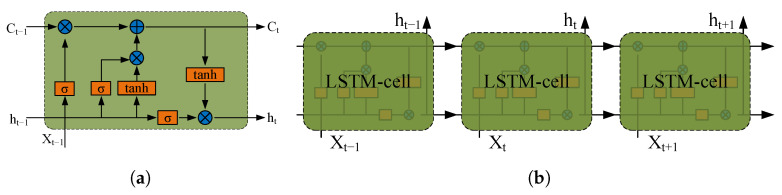
Efficiency map. (**a**) LSTM cell. (**b**) LSTM model.

**Figure 10:**
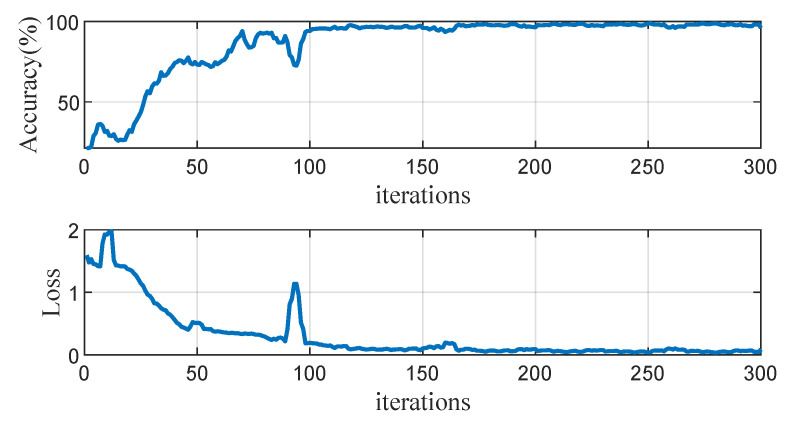
Training progress.

LSTM is widely used for the prediction and classification of time-series or sequence data, e.g., ECG [31,32], sleep apnea [33], and human activities [34]. As shown in Figure 9a, a typical LSTM cell has a memory cell, an input gate, and a forget gate in addition to the hidden state in traditional recurrent neural networks [33,35]. Considering the low amount of training data, we use the dropout technique to randomly drop 20% of the weights during the training process. The LSTM model is shown in Figure 9b. The number of memory cells is set to 100 × 3 and the initial learning rate is set to 0.01. The training process was run for 50 epochs and the training data were shuffled at each epoch to maximize the representability and minimize the variance. The training processes were implemented in MATLAB 2021b. Figure 10 shows the training accuracy and loss functions during the process using the raw sequence data. After training for 100 iterations, the accuracy gradually converges to the final value of about 98%.

## 6. PSO Optimization

In this section, we use the trained LSTM network to evaluate the performance of different weighting parameters and search for the optimal parameters through PSO.

### Fitness Function

The fitness function of optimization consists of two parts: the traditional cost function of MPDMC and the driving feeling. The former is similar to Equation (20), and the latter is generated by the trained LSTM. Notably, the traditional cost function in the fitness function is calculated by the response of the DDEV simulation rather than a simple model of DPDMC. In this, the power loss is calculated by the difference between the input power and the output power. Furthermore, the control accuracy is obtained by the error in the reference and feedback for the longitudinal velocity, the yaw rate, and the lateral displacement.

(21) $$\begin{array}{cc} f= & \lambda_{v}\int C_{v}dt+\lambda_{r}\int C_{r}dt+\lambda_{\beta }\int C_{\beta }dt\\ \; & +\lambda_{l}\int ||\left(P_{in}-P_{out}\right){\left|\right|}^{2}dt+\lambda_{d}\left|\right|D_{ref}-L\left(T_{e}^{\Sigma }\right){\left|\right|}^{2} \end{array}$$

where $\lambda_{d}$ is the weighting parameter of the driving feeling, $D_{ref}=1,2,3$ is the driving feeling command from drivers, $T_{e}^{\Sigma }=\left[T_{e}^{1},T_{e}^{2},\cdots T_{e}^{K}\right]$ is the time sequence of electric torque for in-wheel PMSM, and L(·) is the classification function of the trained LSTM neural network:

(22) $$L\left(T_{e}^{\Sigma }\right)=\left\{\begin{array}{cc} \; & 1\;(\mathrm{if}\;\mathrm{labeled}\;\mathrm{as}\;’\mathrm{Eco}’)\\ \; & 2\;(\mathrm{if}\;\mathrm{labeled}\;\mathrm{as}\;’\mathrm{Nor}’)\\ \; & 3\;(\mathrm{if}\;\mathrm{labeled}\;\mathrm{as}\;’\mathrm{Spt}’) \end{array}\right.$$

## 7. Evaluation

### 7.1. Settings

To validate the effectiveness of the proposed model of predictive direct motion control, numerical simulations were conducted using MATLAB and CarSim software. The standard D series SUV in CarSim was selected as the control object for the simulations, and the key parameters of the vehicle are listed in Table 1. The verification method has two MPDMCs, where the subscript 0 represents those that have not undergone parameter optimization, and the subscript ’opt’ represents those that have undergone parameter optimization. The proposed control strategy will be validated under the following two scenarios:

**A. Acceleration and a double lane change:** The vehicle will first decelerate from 70 km/h to 50 km/h and then accelerate to 80 km/h. The vehicle will travel on a double line change condition, thereby simultaneously verifying the vehicle’s performance in both longitudinal and lateral directions. We analyzed three driving modes to validate the fuzzy evaluation based on the trained LSTM.

**B. Under different road adhesion coefficients with a double lane change:** The vehicle operates under different road adhesion coefficients (μ=[0.3,0.4,0.5]). This case focuses on analyzing the control performance specifically in relation to slip rates, rather than considering different driving modes. Thus, we omit the ‘Eco’ and ‘Spt’ driving modes.

The weights are set to constant values (0.1, 0.2, 10, and 5) for all cases based on prior experience. For different cases, the optimal weights need to be optimized separately.

### 7.2. Case A

#### 7.2.1. “Nor” Mode

**Figure 11:**
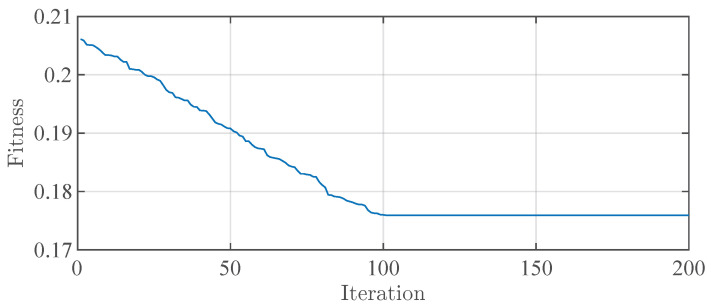
The cost function in iterations.

**Figure 12:**
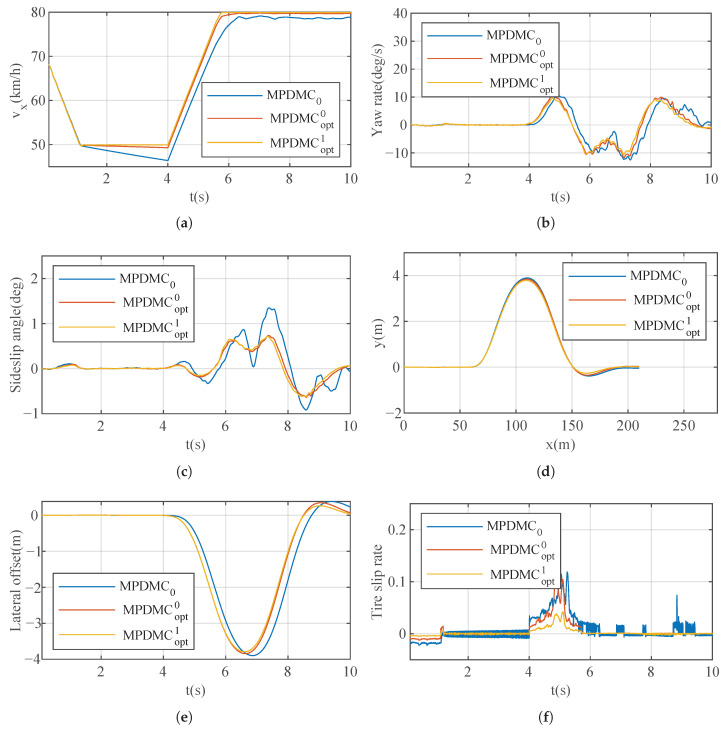
Results of Case A for the “Nor” mode. (**a**) Longitudinal velocity. (**b**) Yaw rate. (**c**) Sideslip angle. (**d**) Track. (**e**) Lateral offset. (**f**) Tire slip rate.

The cost function graph is shown in Figure 11. Within 200 iterations, the cost function gradually decreases from 0.205 to 0.175. It reaches the optimal value around 100 iterations and remains unchanged at 0.175 thereafter. The optimized weights used are [2.0 0.5 8 20], determined through the optimization process to achieve faster longitudinal and lateral responses in this particular scenario. The simulation results are illustrated in Figure 12. In Figure 12a, the vehicle, under MPDMC_opt_, achieves precise tracking of the desired longitudinal velocity with zero error. However, MPDMC_0_ exhibits a noticeably inferior control performance compared to MPDMC_opt_. Particularly, at times of 4 s and 6 s,x when the vehicle begins the double lane change, the nonoptimized weight coefficients in MPDMC_0_ fail to effectively balance the coupling relationship between longitudinal and lateral motion variables. Figure 12b,c depict the vehicle’s yaw rate and sideslip angle, respectively. Significant fluctuations are observed under MPDMC_0_, whereas MPDMC_opt_ ensures a more stable lateral stability. Figure 12d,f show the vehicle’s trajectory and lateral position error, indicating a slightly larger lateral position error in MPDMC_0_ compared to MPDMC_opt_. In Figure 12e, the tire longitudinal slip rate is presented, and it is evident that the fluctuations in the tire slip ratio are significantly reduced under MPDMC_opt_ compared to MPDMC_0_. The comparison of simulation results highlights the substantial influence of the weight coefficients on both the longitudinal and lateral motion control of the vehicle. Through parameter algorithm optimization, MPDMC_opt_ achieves optimal performance in longitudinal velocity control and lateral stability control, thus confirming the necessity and effectiveness of the parameter optimization algorithm. The method MPDMC${}_{\mathrm{opt}}$ with superscripts “0” and “1” denotes model predictive control without and with resistance, respectively. The simulation results of MPDMC${}_{0}$, MPDMC${}_{\mathrm{opt}}^{0}$, and MPDMC${}_{\mathrm{opt}}^{1}$ provide clear evidence that drag plays an important role in vehicle dynamics. Specifically, the omission of drag observed in MPDMC${}_{\mathrm{opt}}^{0}$ causes high-frequency oscillations in the predicted behavior of the vehicle, which is evident in the graph related to the tire slip ratio. However, after the drag was included in MPDMC${}_{\mathrm{opt}}^{1}$, we observed a significant reduction in these high-frequency oscillations. This finding further verifies the importance of considering drag in vehicle models for accurately predicting and controlling vehicle behavior.

#### 7.2.2. “Spt” and “Eco” Mode

**Figure 13:**
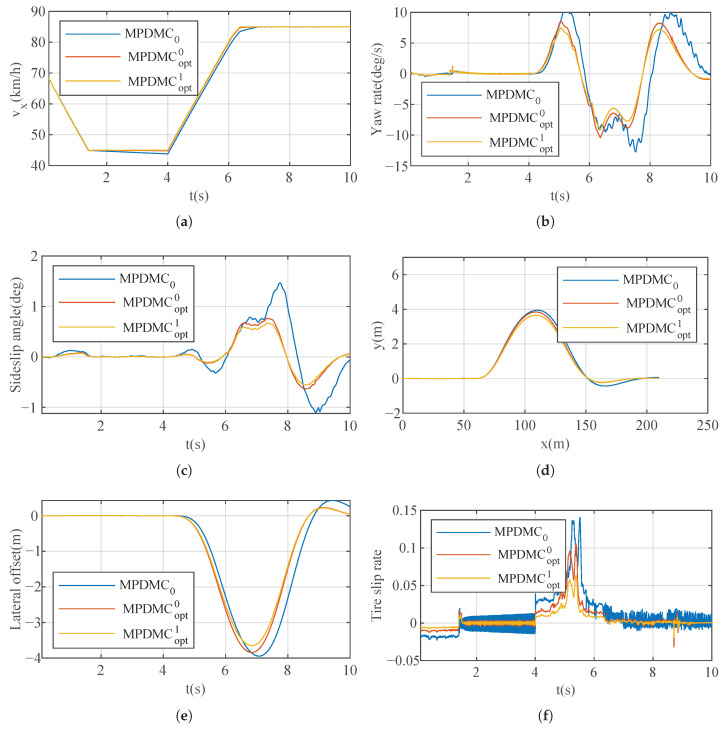
Results of Case A for the “Spt” mode. (**a**) Longitudinal velocity. (**b**) Yaw rate. (**c**) Sideslip angle. (**d**) Track. (**e**) Lateral offset. (**f**) Tire slip rate.

**Figure 14:**
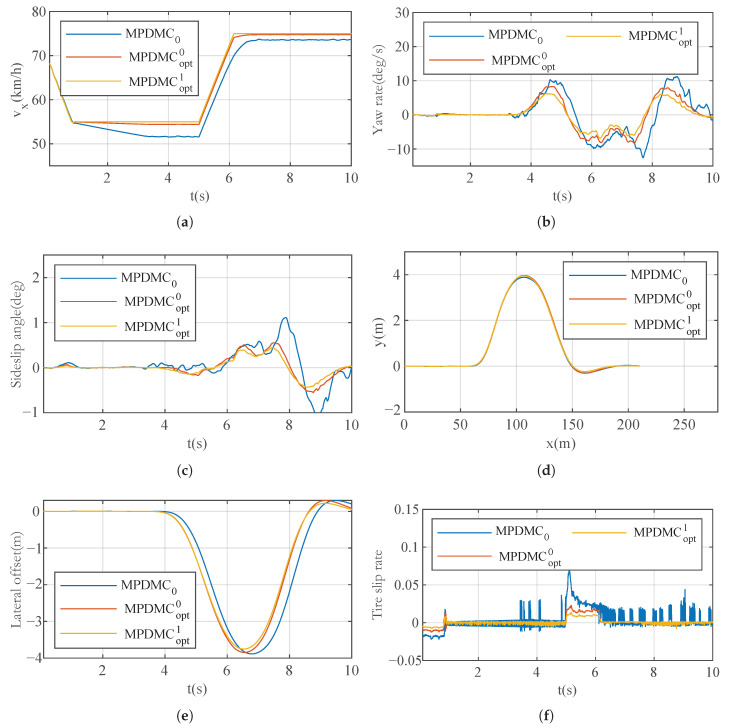
Results of Case A for ’Eco’ mode. (**a**) Longitudinal velocity. (**b**) Yaw rate. (**c**) Sideslip angle. (**d**) Track. (**e**) Lateral offset. (**f**) Tire slip rate.

The simulation results for the “Spt” (sport) mode and “Eco” (economy) mode are illustrated in Figure 13 and Figure 14, respectively. In comparison to the “Nor” (Normal) mode, the “Spt” mode showcases a more aggressive approach to acceleration and deceleration, aiming to achieve a faster response speed. This is evident in the higher slide slip rate and tire slip rate, reaching up to 0.8 and 0.12 radians, respectively.

On the contrary, the “Eco” mode exhibits a more balanced approach, considering that higher acceleration levels result in greater energy consumption. Consequently, the corresponding slide slip rate and tire slip rate in “Eco” mode are lower, reaching a maximum of 0.5 and 0.02 radians, respectively.

### 7.3. Case 2

**Figure 15:**
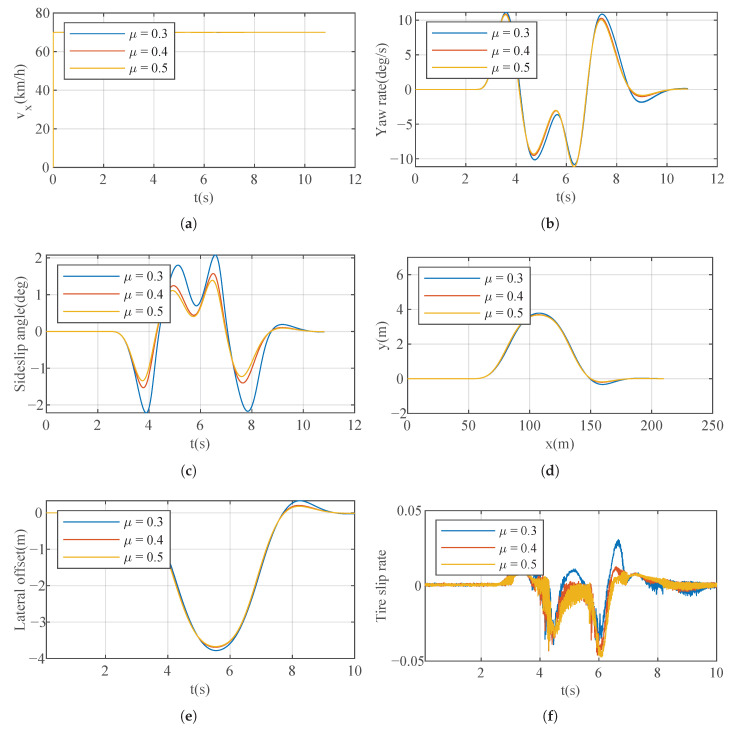
Results of Case 2. (**a**) Longitudinal velocity. (**b**) Yaw rate. (**c**) Sideslip angle. (**d**) Track. (**e**) Lateral offset. (**f**) Tire slip rate.

This part of the simulation is primarily aimed at validating the optimization effects of the parameter optimization algorithm under different road adhesion conditions represented by the coefficient μ. The simulation results are depicted in Figure 15. The optimized weights used were [6.3 0.4 6 2], selected after a process to optimize the slide slip rate, the tire slip rate, and the driving feeling for this specific situation. Figure 15a showcases the vehicle’s performance in low-μ longitudinal motion. Through the parameter optimization algorithm, the vehicle maintains the same longitudinal velocity response across three different μ values. Figure 15b,c demonstrate the validation of the vehicle’s lateral stability under low-μ conditions. Although there is a slight decline in lateral stability when μ = 0.3, overall, the vehicle manages to maintain a consistent lateral control. In Figure 15d,e, the vehicle’s trajectory and lateral position errors remain the same for μ = 0.4 and μ = 0.5, with minimal fluctuations observed at μ = 0.3. In Figure 15f, the tire longitudinal slip ratio similarly reflects an increase at μ = 0.3 but still within a stable range. It is evident that through the proposed parameter optimization algorithm, not only can the vehicle maintain stable motion under low-μ conditions, but it can also eliminate the impact of different μ values, ensuring the vehicle consistently maintains optimal performance in motion control.

## 8. Conclusions

A novel motion control strategy for distributed drive electric vehicles (DDEVs) called model predictive direct motion control (MPDMC) was presented in this paper, which aimed to simultaneously control the longitudinal and lateral motion of the vehicle while considering efficiency and the driving feeling. First, we analyzed the dynamic model of the vehicle, taking into account both the vehicle body and the in-wheel motors. Then, we proposed MPDMC conducted on a single CPU to directly follow the driver’s commands by generating voltage references with a minimum cost function, which consisted of the longitudinal velocity, yaw rate, lateral displacement, and efficiency. The weighting parameters of the cost function were extensively analyzed, and an optimization algorithm based on particle swarm optimization (PSO) was introduced to determine the optimal parameter values. Third, the driving feeling was considered by evaluating it using a trained LSTM network. The LSTM network provided labels for the response under different weighting parameters, classifying them as “Nor”, “Eco”, and “Spt”. The accuracy of the trained LSTM was up to 98 percent. Finally, the performance of the optimized MPDMC was evaluated through extensive simulations conducted using MATLAB and CarSim software. After 100 iterations of optimization, the cost function under one specific work condition was improved from 0.205 to 0.176, an improvement of 14.1 percent. Typical scenarios under different driving feelings were considered for evaluation. The results demonstrated that the optimized MPDMC outperformed the baseline methods, achieving a better overall performance with a faster response, lower slideslip rate, and lower tire slip rate. In the future, we will study the simplification of neural network training under all working conditions and a method to optimize these conditions quickly.

## Figures and Tables

**Table 1:** Parameters of PMSM and vehicles.

Symbol	Quantity	Value
$m_{v}$	Vehicle mass	1152 kg
*R*	Tire radius	0.35 m
$l_{f}$	Center to front axle distance	1.050 m
$l_{r}$	Center to rear axle distance	1.569 m
$d_{f}$	Distance between front wheels	1.565 m
$d_{r}$	Distance between rear wheels	1.565 m
$c_{f}$	Front tire cornering stiffness	79,240 N/rad
$c_{r}$	Rear tire cornering stiffness	87,002 N/rad
*p*	Poles	4
$R_{s}$	Stator resistance	34.3 mΩ
$L_{d}$	D-axis inductance	0.72 mH
$L_{q}$	Q-axis inductance	1.79 mH
$\psi_{f}$	Flux linkage	0.164 Wb
$J_{w}$	Wheel inertia	59.6 $\times \;10^{-6}$ kg·m${}^{2}$
$\omega_{m}$	Rated speed	2850 rpm
$T_{e}$	Rated torque	60 N·m

## Data Availability

Not applicable.
